# Whole-Genome Sequencing of Adenovirus Genotypes and Clinical Implications in Pediatric Patients

**DOI:** 10.3390/v17111480

**Published:** 2025-11-06

**Authors:** Lorena Forqué, Valeria Fox, Rossana Scutari, Martina Mastropaolo, Pietro Merli, Velia Chiara Di Maio, Vanessa Fini, Giulia Linardos, Luana Coltella, Stefania Ranno, Cristina Russo, Alberto Villani, Carlo Federico Perno, Luna Colagrossi

**Affiliations:** 1 Multimodal Laboratory Medicine, Bambino Gesù Children’s Hospital, IRCCS, 00165 Rome, Italy; lforque@tauli.cat (L.F.); rossana.scutari@opbg.net (R.S.); martina.mastropaolo@opbg.net (M.M.); carlofederico.perno@opbg.net (C.F.P.); 2 Department of Hematology/Oncology, Cell and Gene Therapy, Bambino Gesù Children’s Hospital, IRCCS, 00165 Rome, Italy; pietro.merli@opbg.net; 3 Microbiology and Diagnostic Immunology Unit, Bambino Gesù Children’s Hospital, IRCCS, 00165 Rome, Italy; veliachiara.dimaio@opbg.net (V.C.D.M.); vanessa.fini@opbg.net (V.F.); giulia.linardos@opbg.net (G.L.); luana.coltella@opbg.net (L.C.); stefania.ranno@opbg.net (S.R.); cristina.russo@opbg.net (C.R.); luna.colagrossi@opbg.net (L.C.); 4 General Pediatric and Infectious Disease Unit, Pediatric Emergency Medicine, Bambino Gesù Children’s Hospital, IRCCS, 00165 Rome, Italy; alberto.villani@opbg.net

**Keywords:** HAdV, WGS, pediatric infection

## Abstract

Human adenoviruses (HAdV) comprise more than 100 genotypes with species-specific differences in tropism and immune response and can cause severe infections in immunocompromised patients. This study aimed to characterise the HAdV species involved in pediatric infections to assess their clinical impact and guide future therapeutic strategies based on AdV-specific T-cell responses. Between January and October 2024, 595 pediatric HAdV diagnoses were made at the Bambino Gesù Children’s Hospital (Rome), and whole-genome sequencing was performed on 60 samples. Most patients (91.7%) were hospitalised, including both immunocompetent (75%) and immunocompromised (25%) children. Gastrointestinal and respiratory symptoms were more common in immunocompetent patients, whereas immunocompromised patients experienced longer hospitalisations and persistent viral infections. Species F (F41) was most prevalent (63.3%), especially among immunocompetent patients, while species C and A predominated in immunocompromised children, with species A associated with severe disease. Viral loads were significantly higher for species F than for species A and C, independent of immune status. Co-infections were frequent (63.3%), with species C particularly linked to them. In conclusion, HAdV distribution differed by immune status, with species F predominating in immunocompetent children and species C and A more common in immunocompromised patients. Whole-genome sequencing may enhance surveillance, enable earlier diagnosis, and support the development of genotype-specific immunotherapies.

## 1. Introduction

Human adenoviruses (HAdVs) are highly contagious pathogens with significant clinical importance, particularly in the pediatric population. These viruses are a major public health concern, with nearly 80% of individuals experiencing at least one episode of HAdV infection by the age of six. HAdVs are a frequent cause of community-based pathologies, including but not limited to diarrhoea, acute respiratory infections, conjuntivitis, and urinary tract infections [1]. Immunological status is a key influencing factor for patients’ prognosis. Indeed, HAdV infections are typically self-limiting in immunocompetent individuals, while they can be severe and life-threatening in immunocompromised patients. This increased severity is particularly relevant in patients undergoing hematopoietic stem cell transplantation (HSCT) or solid-organ transplantation, where HAdV can reactivate from sites of latency, including lymphoid tissues mainly associated with intestinal mucosa [2].

There are currently seven species of HAdV (A–G), with 51 serotypes and 116 genotypes identified and recognized by the International Committee on Taxonomy of Viruses (ICTV) [http://hadvwg.gmu.edu, accessed on 13 February 2025]. This high genetic variability is mainly due to recombination processes that occur only between HAdV genotypes of the same species and in regions of high sequence homology. This important mechanism of viral evolution favors the emergence of new strains that may have different fitness and pathogenicity [3], as well as different sensitivity to the control by the immune system. In the next years, the number of identified genotypes is expected to rise due to the increased use of next generation whole-genome sequencing (WGS), a unique system able to better characterize the genome of HAdV strains [4,5,6].

The great genetic diversity of HAdV allows for a broad tropism through interactions with different cellular receptors, resulting in a wide range of clinical manifestations depending on the species and genotype involved [7]. For instance, in the pediatric population, the F41 genotype typically causes self-limiting diarrhea, while some HAdV-C genotypes, such as HAdV1, HAdV2, and HAdV89, cause severe infections with life-threatening implications in young children and immunocompromised patients [3]. In the same way, HAdV-B genotypes (e.g., B3, B7, B14, B21, B55) are frequently associated with severe pediatric respiratory illnesses requiring hospitalization and community-acquired pneumonia in adults [6,8]. Therefore, accurate identification of the HAdV genotype is clinically relevant, as it facilitates stratification of patients according to the risk of developing more severe disease and guides treatment strategies, remembering that this relatively specific distinction in terms of target organs of HAdV is less evident in cases of heavy immunosuppression, when HAdV strains of various serotypes and species can cause disease at different sites and organs. Another important reason for the genotypic characterization of HAdV, innovative therapy, is based on therapeutic attempts based on the infusion of adenovirus-specific T cells generated in vitro using HAdV serotype-specific peptides, to boost the patient’s immune response [9,10,11].

The identification of genotypes is currently limited by the routine diagnostic approaches. These methods allow the detection of viral genomic DNA or antigens, but are unable to differentiate specific HAdV genotypes or identify novel and/or potentially virulent genotypes arising from viral recombination. For this reason, cost-effective and time-efficient WGS methods that can be integrated into clinical adenovirus characterization are needed to identify key genetic determinants of virulence and fitness, as well as key determinants of host susceptibility. This also requires the optimisation of extraction protocols that allow the sequencing of HAdV directly from biological samples, as the high proportion of human DNA in these samples remains a major obstacle to successful whole-genome sequencing.

This study aimed to characterize the HAdV species involved in pediatric infection, by optimized sequencing protocols from direct biological samples, in order to understand their potential impact on disease progression and to guide the selection of therapies based on HAdV-specific T-cell responses.

## 2. Materials and Methods

### 2.1. Population Study

From 1 January to 31 October 2024, a total of 20,258 samples (including stool, respiratory, and blood samples) were tested for HAdV at Bambino Gesù Children’s Hospital (Rome, Italy), resulting in 595 new HAdV molecular diagnoses among pediatric patients (aged 0–18 years). Among them, 60 positive samples (53 stool and 7 nasopharyngeal aspirate) were selected for HAdV whole-genome sequencing (WGS), according to the following inclusion criteria: viral load > 1000 cp/mL or <29 cycle threshold (Ct) values in samples from patients < 18 years of age. Demographic and clinical data were collected retrospectively and anonymously for all patients.

To assess the potential impact of co-infections on disease severity, all positive microbiological results per patient, performed within 2 weeks before and 2 weeks after the date of the sequenced adenovirus sample, were collected.

To calculate the duration of adenovirus infection, both overall and gastrointestinal, we defined two variables: Time of positivity for HAdV and time of positivity for gastrointestinal HAdV.

Time of positivity for HAdV was defined as the period during which adenovirus was detectable by molecular methods on any type of sample (respiratory, gastrointestinal, blood, and urine samples). Time of positivity for gastrointestinal HAdV was defined as the period during which adenovirus was detectable by molecular methods only on stool samples.

### 2.2. Molecular Methodology

The respiratory samples were routine clinical specimens collected using nasopharyngeal swabs in Universal Transport Medium (UTM) or nasopharyngeal aspirate. Respiratory viruses were assessed using the Allplex Respiratory Panel assay (Seegene Inc., Seoul, Republic of Korea), a multiplex one-step real-time RT-PCR system that simultaneously detects 16 different respiratory viruses, including adenovirus. This system includes extraction, amplification, detection, and analysis of samples and provides cycle threshold (Ct) values for all positive targets. A sample was considered positive if the Ct value was ≤42.

Stool and blood samples were extracted using the QIAsymphony DSP Virus/Pathogen Midi Kit and QIAsymphony DSP DNA Mini Kit, respectively. For HAdV detection, 10 µL of extracted DNA were used for quantitative Real Time PCR (Argene, bioMerieux, Marcy l’Etoile, France), in accordance with the manufacturer’s instructions. The results were expressed in copies per milliliter (copies/mL).

### 2.3. Viral DNA Extraction and Whole-Genome Sequencing

Whole genome sequencing was performed on 60 HAdV strains to characterize the circulating genotypes in our pediatric population. The samples were selected randomly based on viral load. The samples were selected randomly based on viral load. The viral load cutoff of >1000 copies/mL for stool specimens and the Ct value < 29 for respiratory samples were selected based on our laboratory’s experience and previous data, which indicate that these thresholds ensure sufficient viral DNA concentration for reliable sequencing.

A home-made pre-extraction protocol was used to isolate viral genomes and to remove human and bacterial DNA directly from biological samples. Specifically, 1 g of stool and respiratory samples were pre-processed to reduce the viscosity with stool preprocessing device (bioMerieux, Marcy l’Etoile, France) and SLsolution (Copan Italia, Brescia, Italy, in a 1:1 ratio), respectively. Subsequently, the 1.5 mL of each sample (stool or nasopharyngeal aspirate) was centrifuged at 17,000 rpm for 5 min, and the supernatant was filtered through a 0.45 µm filter, to remove human and bacterial DNA. HAdV-DNA was extracted using the automatic extractor EZ2 (Qiagen BioRobot EZ2, Qiagen, Hilden, Germany) with the appropriate viral extraction kit (EZ1&2 Virus Mini v2.0 kit, Qiagen, Hilden, Germany), following the manufacturer’s instructions and setting the elution volume at 60 µL from 400 uL of sample. Extracted DNA was quantified using Qubit Instrument (Qubit^®^ dsDNA HS Assay Kits, Thermo Fisher Scientific, Waltham, MA, USA) to verify that the extraction was successful.

Next Generation Sequencing library preparation was performed according to the manufacturer’s protocol with the DNAprep kit (Illumina, San Diego, CA, USA). Prepared libraries were sequenced with an Illumina MiSeq sequencing platform.

### 2.4. Interpretation of Data and Statistical Analysis

Descriptive statistics are expressed as median values and interquartile range (IQR) for continuous data and number (percentage) for categorical data. Statistical comparisons were performed using Fisher’s exact and Mann–Whitney tests for categorical and continuous variables, respectively. A *p*-value less than 0.05 was considered statistically significant. Data were analysed using statistical software package SPSS (v32.0; SPSS Inc., Chicago, IL, USA).

### 2.5. Bioinformatics Methods

Raw reads obtained from sequencing were trimmed for adapters and quality using Fastp (v0.23.2) [12,13], by checking the quality before and after filtering with FastQC (v.0.11.9) [14] and MultiQC (v1.18) [15]. Reads mapping to the human genome (GRCh38) were removed using bbsplit (BBTools, https://sourceforge.net/projects/bbmap/ (accessed on 19 February 2025)). The remaining clean reads were transformed back to paired end reads using reformat (BBTools). Kraken2 (v2.1.3) [16] was then used with parameter--confidence 0.5 with the standard database to determine the taxonomic classification and screen for remaining human reads, for which a second step of host decontamination was performed. Trimmed and decontaminated reads were *de novo* assembled by Shovill (v1.1.0) [17]. A custom approach to initially screen for putative genotype was performed using the obtained contigs and blasting them against three custom databases, constructed with 112 fiber, penton and hexon sequences, obtained from NCBI database and the HadV Working Group website (http://hadvwg.gmu.edu**/** (accessed on 20 February 2025)). The genotypes with the highest nucleotide identity and query coverage were then used as reference sequences for mapping with BWA-mem (v0.7.17) [18]. After mapping, Single Nucleotide Polymorphysm (SNP) variants were called with freebayes (v1.3.2) [19] and all SNPs having a minimum read frequency of 40% and depth of ≥10 were used to construct a consensus sequence using the vcf_consensus_builder tool (https://github.com/peterk87/vcf_consensus_builder (accessed on 20 February 2025)).

### 2.6. Phylogenetic and Recombination Analysis

Whole genome consensus sequences were used for phylogenetic analysis. Sequences were aligned using MAFFT (v7.490) using default settings and manually inspected using Aliview (v.2021), including also genotype-specific reference genomes, retrieved by NCBI database (Supplementary Table S1). The final alignment was used as input for phylogenetic analysis by Maximum Likelihood (ML) method, performed by IQTREE (v1.6.12) [20] with 1000 bootstrap replicates, using the best-fit nucleotide substitution model inferred by ModelFinder. Phylogenetic tree obtained was then annotated using iTOL (v7) [21].

## 3. Results

### 3.1. Patient’s Clinical Characteristics

Among the 60 selected patients, 45 (75%) were immunocompetent and characterized by a median age of 1.4 years (IQR: 0.74–2.61), while 15 (25%) were immunocompromised with a median age of 12.5 years (IQR: 4.99–15.85). Most of them (55/60, 91.7%) were hospitalized, with similar hospitalization rates among immunocompetent and immunocompromised patients (95.6% vs. 80.0%, *p* = 0.094). However, the immunocompromised have shown a longer length of hospitalization (median 103 days, [IQR: 11.0–149.0] vs. 3 days, [IQR: 2.0–5.0], *p* < 0.001, Table 1).

At the time of HAdV diagnosis, diarrhea (72.4%) was the most frequently reported gastrointestinal symptom, followed by vomiting (56.9%). Among respiratory symptoms, pharyngitis (34.4%) and rhinitis (17.2%) were the most common (Table 1). When focusing on the immunological status of patients, gastrointestinal and respiratory symptoms were more prevalent in immunocompetent than immunocompromised patients (91.1% vs. 69.2%, *p* = 0.066 and 53.3% vs. 23.1%, *p* = 0.066, respectively). This trend was also observed when the symptoms were taken into consideration individually: diarrhea (77.8% vs. 53.8%, *p* = 0.156), vomiting (60.0% vs. 46.2%, *p* = 0.526), dehydration (60.0% vs. 7.7%, *p* = 0.001), rhinitis (20.0% vs. 7.7%, *p* = 0.301), pharyngitis (42.2% vs. 7.7%, *p* = 0.023), and cough (13.3% vs. 7.7%, *p* = 1.000) (Table 1).

Fever was the only systemic symptom that could be consistently assessed in this cohort. When analyzed, a higher incidence was observed in immunocompromised patients compared to immunocompetent individuals (53.8% vs. 44.4%, *p* = 0.549), although the difference was not statistically significant.

However, the persistence of viral infection was significantly longer in immunocompromised than in immunocompetent patients. This difference was observed both when considering the presence of the virus in any body compartment (respiratory, gastrointestinal, and systemic) (median 1 day, [IQR 1–4] vs. 58 days, [IQR 17–66]; *p* < 0.001) and when exclusively assessing viral presence in stools (median: 2 days, [IQR 1–18] vs. 32 days, [IQR 17–63]; *p* = 0.037, Table 1).

### 3.2. Genotypes Distribution and Characteristics

The HAdV genome sequencing revealed that the majority of HAdV belonged to species F (63.3%, 38/60), followed by species C (25.0%, 15/60), A (8.3%, 5/60) and B (3.3%, 2/60). All species F strains were identified as genotype F41 (100.0%, 38/38). In species C, the most common genotypes were C89 (46.7%, 7/15), followed by C1 (26.6%, 4/15), C108 (20.0%, 3/15), and C5 (6.7%, 1/15). Within species A, genotypes A31 (60.0%, 3/5) and A12 (20.0%, 1/5) were detected, while one strain (20.0%, 1/5) remained untyped due to a low sequencing depth. In species B, both strains were identified as genotype B114 (100%, 2/2).

**Figure 1:**
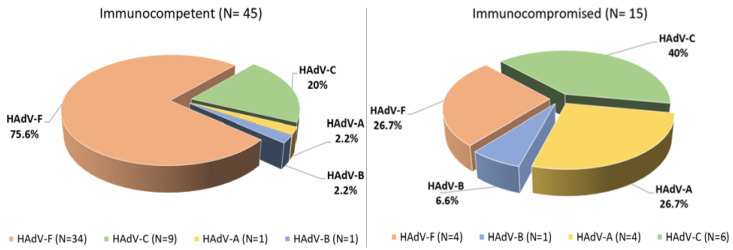
Distribution of HAdV species in immunocompetent and immunocompromised patients.

The HAdV species observed showed a different distribution between immunocompromised and immunocompetent patients. Specifically, species F was more prevalent in immunocompetent patients (75.6%, 34/45 vs. 26.7%, 4/15; *p* = 0.001), while in immunocompromised patients the most prevalent species were C (40%, 6/15 vs. 20%, 9/45; *p* = 0.169) and A (26.7%, 4/15 vs. 2.2%, 1/45; *p* = 0.012) (Figure 1). The latter two have been previously associated with severe infections, particularly in children and immunocompromised individuals. It should be noted that the median age of immunocompetent patients was significantly lower than that of immunocompromised patients, so it is possible that differences in the distribution of adenovirus species are not exclusively related to immune status, but rather to the combined effect of both factors.

Furthermore, the length of hospital stay was significantly shorter in patients infected with species F (median 3 days [IQR: 2.0–6.0]) compared to those infected with species A (median 31 days, [IQR: 6.0–121.0], *p* = 0.025) and species C (median 5 days, [IQR: 4.0–101.0], *p* = 0.054).

These findings are consistent with the results observed for time to HAdV positivity, where species F had a shorter median time of 4 days (IQR: 1–17), compared to species A (median: 35 days, IQR: 19–103; *p* = 0.018) and species C (median: 59 days, IQR: 7–65; *p* = 0.135). The shorter durations of hospitalization and viral detection associated with species F likely reflect its predominance among immunocompetent patients. By contrast, species A, B and C were more frequently detected in immunocompromised individuals, who are predisposed to prolonged viral persistence and hospitalization (Figure 1).

In line with these observations, multivariable analysis showed that patients infected with species A, B, or C had a 13-fold higher risk of presenting with fever compared to those infected with species F (Table 2).

These findings underscore the significant impact of immunosuppression on viral reactivation and persistence, highlighting the need for targeted surveillance and management strategies for this vulnerable population.

For the viral load analysis, eight samples were excluded due to differences in quantification methods. For five samples, only Cycle threshold (Ct) values for respiratory samples were available (N = 5, median Ct = 17.16), while corresponding stool adenovirus Ct values were not determined. For three samples, viral load in fecal samples was quantified using a different PCR assay that reported results in copies per milliliter (cp/mL) (N = 3, median = 81,187,200 cp/mL), rather than Ct values. In contrast, Ct values were available for 52 samples (N = 50 fecal, N = 2 fecal from patients with ARF sequencing).

**Figure 2:**
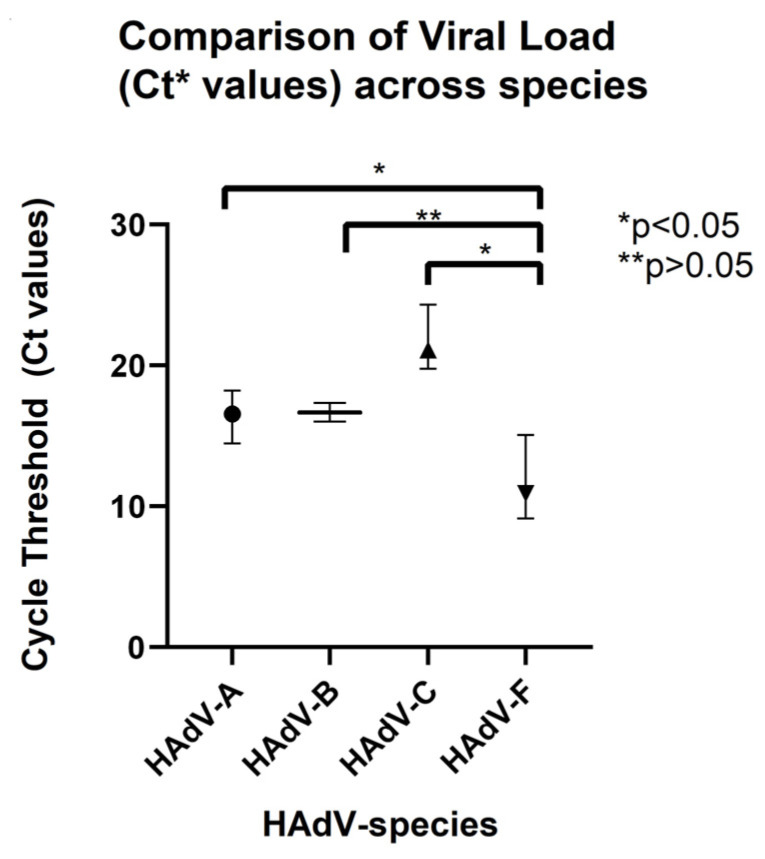
Comparison of viral load (VL) according to species.

When we analysed the viral load at enrolment (N = 52), we found that the peak viral loads were significantly higher (corresponding to lower Ct values) for species F (median Ct 10.9 [IQR: 9.2–15.0]) than for species A (median Ct 16.6 [IQR: 15.0–17.8], *p* = 0.022) and species C (median Ct 21.0 [IQR: 20.3–24.3], *p* < 0.001) (Figure 2). This difference may be attributed to the specific tropism of species F for the gastrointestinal tract, where viral replication results in higher viral loads in the stool samples compared to those of other species.

In general, immunocompetent patients showed lower Ct values (indicating higher viral loads) compared to immunocompromised patients (median Ct: 12.5 vs. 17.0, respectively, *p* = 0.04). However, no significant differences in Ct values were observed when comparing immunocompetent and immunocompromised patients within each species, except for species C, which showed a higher viral load (lower Ct values) in immunocompetent patients (Table 3). Nevertheless, the small sample size prevents any firm conclusion.

### 3.3. Co-Infections

In our cohort, co-infections (gastrointestinal, respiratory, urinary or systemic) with other viruses and/or bacteria were detected in 63.3% (38/60) of patients, with no significant difference between immunocompetent (28/45) and immunocompromised (10/15) patients (62.2% vs. 66.6%, respectively, *p* = 1.000).

When analyzing the types of co-infections individually, gastrointestinal co-infections were the most prevalent (41.6%, 25/60), followed by respiratory (21.6%, 13/60), systemic (20.0%, 12/60) and urinary (10.0%, 6/60). The distribution of co-infections was similar between immunocompetent and immunocompromised patients, except for systemic co-infections, which were significantly more frequent in the latter group (6.6%, 3/45 vs. 60.0%, 9/15 *p* < 0.001).

Gastrointestinal co-infections were primarily associated with enteropathogenic Escherichia coli (EPEC) carrying the eaeA gene, detected in 52.0% (13/25) of cases, while Norovirus was the most frequently detected viral pathogen, present in 16.0% (4/25) of gastrointestinal co-infections. No significant differences in hospitalization duration were observed between patients with and without gastrointestinal co-infection (*p* = 0.468)

Respiratory co-infections were predominantly linked to Rhinovirus, identified in 61.5% (8/13) of cases. Systemic co-infections were primarily caused by Herpesviruses, including HHV-6, EBV, and CMV, which together accounted for 75% of systemic co-infections. Urinary co-infections were evenly distributed between bacterial pathogens (predominantly Enterobacteriaceae and *Enterococcus faecalis*) and BK virus reactivation.

Regarding the distribution of different Adenovirus species in mono- and co-infections, species C was predominantly detected in co-infections (73.3%, 11/15 of co-infections vs. 26.7%, 4/15 of mono-infections, *p* = 0.027). In contrast, the distribution of species F was comparable between mono- and co-infections, with no statistically significant difference (55.2%, 21/38 vs. 44.8%, 17/38, *p* = 0.492).

When analyzing viral load (Ct values), no significant differences in Adenovirus viral load were observed between mono-infections and overall (bacterial and viral) co-infections (median Ct 14.0 [9.5–16.6] in monoinfections vs. 14.3 [9.7–19.5] in coinfections, *p* = 0.735). These findings remained consistent within each species, suggesting that viral load was not influenced by the presence of additional pathogens.

In the context of gastrointestinal viral co-infections, a trend towards higher HAdV viral loads (lower Ct values) was observed in mono-infections (median Ct 13.5 [9.7–16.7] vs. 16.4 [10.9–21.1] in co-infections). However, this difference was not statistically significant (*p* = 0.296), indicating that adenovirus viral load is not notably influenced by viral co-infection.

### 3.4. Phylogenetic Analysis

**Figure 3:**
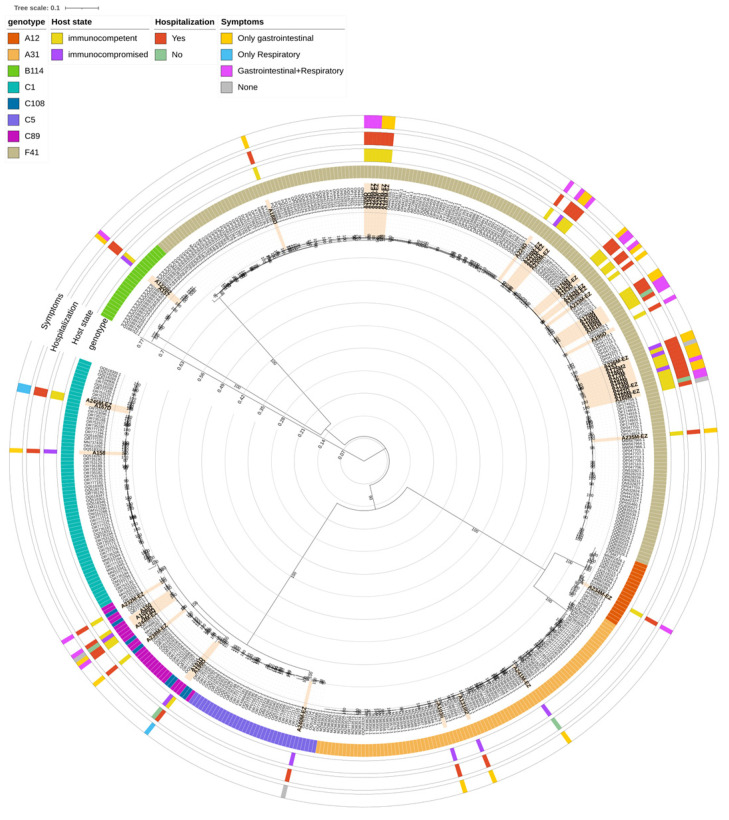
Estimated Maximum Likelihood phylogenetic tree based on the whole-genome sequences of 56 HAdV isolates. The tree was constructed incorporating 426 references of 8 different genotypes downloaded from NCBI database. The phylogeny was estimated with IQTREE using the best-fit model of nucleotide substitution SYM + R10 with 1000 replicates fast bootstrapping. Leaves number represents the sample ID, bootstrap values higher than 90 are shown on branches. Clinical samples obtained in this study are colored orange. Information regarding genotype, host immune state, hospitalization and symptoms are reported as annotations. Tree scale is expressed as nucleotide substitutions per site.

Phylogenetic analysis was conducted on 56 samples (51/56 [91.1%] from stools and 5/56 [8.9%] from nasopharyngeal aspirate) for which a complete genome could be obtained by WGS, incorporating 426 reference sequences belonging to 8 AdV-species and retrieved from the NCBI database (Figure 3). The Maximum Likelihood tree confirmed that the samples clustered with the reference sequences of their assigned genotypes based on WGS analysis, with the only exception of those classified as genotype C108. Notably, the phylogenetic analysis did not show a distinct clustering of C108 sequences; instead, they were interspersed among C89 strains, in a cluster supported by a bootstrap of 100. This may be due to the fact that both C108 and C89 genotypes are recombinant forms derived from the C2 genotype.

Within the C1 genotype, clinical samples clustered in two separate groups: two samples, coming from immunocompetent patients, clustered with reference sequences originating from the USA, including the original C1 reference (GenBank acc. n° AF534906.1), while another sample, from a immunocompromised patient, clustered specifically with one sequence isolated in the USA in 2009 (GenBank acc. N° OQ518262.1). Similarly to C1, genotype C5 clinical sample clustered with sequences originating mainly from the USA in years 2009–2020.

When looking at genotype B114, two clinical samples, from an immunocompromised and immunocompetent patient, respectively, clustered together with seven sequences retrieved from NCBI, which are all originating from Germany in 2023.

Within species A, one A12 sample clustered with sequences coming from different countries (the USA, Canada, Sweden and Kenya), and particularly with a sequence isolated in 2023 in coastal Kenya during an acute gastroenteritis outbreak in children (GenBank acc. N° PQ336882.1). Three samples, identified as genotype A31, clustered with a total of 59 reference sequences of the same species originated from different geographic regions (mainly the USA and Germany, followed by China and Tunisia) from 2006 and 2021.

In the case of genotype F41, a total of 38 sequences were included in the phylogenetic analysis. By looking at the clades already observed in literature [22,23], most of the clinical samples (30/38, 78.9%), clustered with references belonging to L2b clade, which originated from multiple regions (France, Germany, China, Spain, UK, the USA, and Iraq) from 2016 to 2022 (Supplementary Figure S1). This is consistent with what was already observed in literature, where L2b is the most represented of F41 clades. Clade L2a included 7 clinical samples, which clustered with sequences coming from several countries (mainly Germany and Kenya, followed by the USA, China, UK, France, Japan, Netherlands and Sweden) from 2000 to 2023. Lastly, a single clinical sample, from an immunocompetent patient, clustered with reference sequences of clade L1, including the prototype Tak strain (1973, GenBank acc. n° DQ315364.2) and other strains isolated from China, Kenya, France, Germany, Netherlands, Spain and Iraq from 2007 to 2023.

## 4. Discussion

Our study provides whole-genome sequencing data from one of the largest pediatric adenovirus (HAdV) positive cohorts described to date, including both immunocompetent and immunocompromised patients.

One of the most innovative aspects of our study lies in the fact that, while WGS has been increasingly employed in recent years, most studies have mainly focused on adult populations [24,25], or smaller pediatric cohorts [26,27], often limited to specific clinical contexts or outbreaks [3,28,29]. These studies, although extremely valuable, tend to describe highly specific situations, usually within hematopoietic stem cell transplant units, and thus might fail to reflect the overall picture of HAdV specific genotypes circulation outside those contexts. In contrast, our study allowed for a broader snapshot of the circulating adenovirus landscape in a mixed pediatric population, including both immunocompetent and immunocompromised patients, and outside of outbreak contexts.

Moreover, most studies are based on the sequencing of specific targets or partial genomic regions, as obtaining complete genomes is often limited by the need to isolate the virus in cell cultures. Cell culture-based HAdV isolation is not routinely used in diagnostics, and its labour-intensive nature likely limits the application of genotypic characterization in clinical practice. In this study, we describe a pre-sequencing protocol that allows the whole genome to be sequenced directly from stool samples, without the need for prior virus isolation in cell cultures. Therefore, this approach could facilitate broader genomic surveillance, especially in paediatric settings, where stool samples are readily available.

This makes our data more representative of routine clinical practice and community-level viral circulation.

In our cohort, species F was the predominant species (63.3%), and all strains of species F were identified as genotype F41. Other species detected were C (25%), A (8.3%) and B (3.3%), with several genotypes represented within these species, including conventional (C1, C5, A31, A12) and recombinant (C89, C108, B114) strains previously associated with variable pathogenicity. The predominance of HAdV-F41 in our cohort may, at least in part, reflect a sampling bias associated with the predominance of stool-derived specimens, which are known to be enriched for Species F adenoviruses, particularly in pediatric populations.

The distribution of species varied according to immune status: species F predominated in immunocompetent children, while species C and A were more frequent in immunocompromised patients. Although this distribution is consistent with previous reports [30,31,32,33], it is unlikely that some clinical outcomes, such as longer hospital stays in immunocompromised patients, are due solely to HAdV species. Instead, these outcomes are more likely driven by underlying comorbidities that profoundly modulate host–virus interactions. In particular, immunocompromised patients often exhibit delayed viral clearance, which could explain the observed differences in disease severity and recovery time. In this context, co-infections appear to play a limited role compared to the impact of host-related clinical conditions.

Comparable patterns of HAdV circulation have been described in Italy and other European countries, where species F, particularly genotype F41, is most frequently detected among cases of paediatric gastroenteritis, and species C (especially genotypes C1, C2, and C5) is most frequently associated with severe disease in immunocompromised hosts [3,26]. Species B (particularly genotype B3) has also been frequently described in Italian studies, mainly associated with acute respiratory infections [34,35]. In our study, the only two strains detected of species B corresponded to the recently described genotype B114 (2023). The literature on this new genotype remains scarce and is limited to reports published this year in Germany and China, where B114 has been associated with respiratory infections [36,37]. It is believed that this genotype has been circulating for some time but was previously misclassified due to its close phylogenetic similarity to genotype B3 [36].

Interestingly, we also identified genotype A12 in our cohort, a strain that is rarely detected in humans but is increasingly recognised for its potential oncogenic properties demonstrated in animal models [38]. Nonetheless, a surveillance study conducted on wastewater in Italy reported the presence of HAdV-A12 in wastewater samples collected in Milan (Italy) in December 2021, supporting the hypothesis of circulation of this genotype in Italy [39].

Higher viral loads were observed in infections caused by species F, genotype F41, which is consistent with its gastrointestinal tropism and efficient replication in the intestinal mucosa, leading to increased faecal excretion [40]. Coinfections were common, occurring in more than 60% of cases, mainly with gastrointestinal pathogens such as Escherichia coli and norovirus. No significant differences in hospitalization duration were observed between patients with and without gastrointestinal co-infection. Notably, a Danish study also reported norovirus as the most common viral co-infection in fecal samples [41].

The high prevalence of coinfections highlights the complex microbial environment in infected patients, but the independence of adenoviral load from co-pathogens suggests that HAdV could play a primary role in viral replication dynamics. However, the clinical significance of these co-infections remains unclear and warrants further study [42].

Regarding associations between adenovirus genotypes and disease severity, our data did not reveal definitive correlations. This is likely due to the uneven distribution of genotypes within our cohort; for example, all strains of species F were genotype F41 (100%, 38/38), while the non-F species were represented by a small number of cases across multiple genotypes, limiting statistical power for comparisons. This heterogeneity of genotypes, combined with the multifactorial nature of disease severity, including host immune status and comorbidities, makes it difficult to draw firm conclusions at this stage. Larger longitudinal studies with a more balanced representation of genotypes are needed to clarify these relationships.

Another emerging area of interest is the potential for cross-immunity between adenovirus genotypes. CD4^+^ T cells appear to be able to recognize conserved epitopes shared between different genotypes, suggesting that previous infections or adenovirus-based vaccines may confer partial protection against multiple strains [43]. However, the high capacity of adenoviruses to generate new recombinant genotypes makes it difficult to draw firm conclusions about cross-immunity. Further studies like ours, providing a detailed characterization of adenovirus genomes in pediatric infections, are needed to advance the investigation of virus–host interactions and, in the future, to contribute to the development of genotype-based immunotherapies.

Our study has some limitations. Illumina short-read sequencing, although effective for genotyping, was less capable of detecting mixed infections involving multiple adenovirus genotypes in a single sample, particularly relevant in immunocompromised patients prone to reactivations. Furthermore, although the overall sample size was adequate, the limited representation of certain genotypes limits our ability to further investigate potential correlations between specific genotypes, disease severity, and clinical outcomes. However, sample collection is ongoing, and the increasing number of cases will enhance the representativeness and statistical power of future analyses.

In conclusion, our findings expand current knowledge about the epidemiology and clinical correlates of adenovirus infections in pediatric populations. We also describe a novel culture-independent pre-sequencing method for whole-genome sequencing directly from clinical samples, which could facilitate broader genomic surveillance in the future.

In light of the increasing use of high-throughput sequencing and personalized cell therapies, these results underscore the need for larger longitudinal studies to explore the genetic diversity of adenoviruses and their interaction with host immunity. Improved genotype-based diagnostics could facilitate the personalization of treatment strategies, which would particularly benefit immunocompromised children. In fact, adenovirus therapy is primarily symptomatic, and strategies vary based on the severity of the infection and the patient’s immune status. In particular, in pediatric patients who develop adenovirus infection following hematopoietic stem cell transplantation (HSCT), the transplant of virus-specific T lymphocytes is an emerging and promising therapeutic option. However, the epitopes used in laboratory may not be specific to the adenovirus genotype infecting the patient. This could lead to a partial response. Considering that there are more than 100 adenoviruses that differ in tissue tropism, virulence and immune response, knowing the genotype could allow the viral target to be accurately identified and the selection of T lymphocytes specific to the protein epitopes of that genotype to be guided. Furthermore, a curate identification of AdV genotypes could be clinically relevant for stratifying patients according to their risk of developing more severe disease.

We believe that our findings not only contribute to enriching existing knowledge, but also provide a methodological framework for future surveillance and research, while offering important insights into the genomic heterogeneity of HAdVs across various clinical scenarios in children.

In order to develop this topic, further studies are ongoing to study the potential impact of viral mutations on the binding to host receptors, which could influence the virus’s fitness, virulence, and pathogenicity.

## Figures and Tables

**Table 1:** Patients’ characteristics.

	Overall	Immunocompetent	Immunocompromised	*p*-Value $
**Total Samples, N (%)**	60 (100)	45 (75)	15 (25)	
**Male, N (%)**	28 (46.7)	23 (51.1)	5 (33.3)	0.371
**Age, median [IQR] years**	1.96 [0.83–6.03]	1.4 [0.74–2.61]	12.5 [3.99–17.26]	**<0.001**
**Type of samples, N**				
Stool	53	39	14	
Nasopharyngeal aspirate	7	6	1	
**Hospitalization, N (%)**	55 (91.7)	43 (95.6)	12 (80.0)	0.094
**Days of hospitalization, median [IQR]**	4 [2.0–8.0]	3 [2.0–5.0]	103 [11.0–149.0]	**<0.001**
Time of positivity for HAdV, median [IQR] days *	17 [1.0–59.0]	1 [1.0–4.0]	58 [17.0–66.0]	**<0.001**
Time of positivity for gastrointestinal HAdV, median [IQR] days **	28 [13.0–59.0]	2 [1.0–18.0]	32 [17.0–63.0]	**0.037**
Symptoms at HAdV diagnosis ***				
**Gastrointestinal, N (%)**	50 (86.2)	41 (91.1)	9 (69.2)	0.066
Diarrhea	42 (72.4)	35 (77.8)	7 (53.8)	0.156
Vomiting	33 (56.9)	27 (60.0)	6 (46.2)	0.526
Dehydration	28 (48.3)	27 (60.0)	1 (7.7)	**0.001**
**Respiratory, N (%)**	27 (46.6)	24 (53.3)	3 (23.1)	0.066
Rhinitis	10 (17.2)	9 (20.0)	1 (7.7)	0.301
Pharyngitis	20 (34.4)	19 (42.2)	1 (7.7)	**0.023**
Cough	7 (12.1)	6 (13.3)	1 (7.7)	1.000
Fever, N (%)	27 (46.6)	20 (44.4)	7 (53.8)	0.549

IQR, interquartile range. * Median (IQR) was calculated on 25 individuals (12 immunocompetent pts and 13 immunocompromised pts) with more than one HAdV positive sample from any body compartment (respiratory, gastrointestinal, and systemic). ** Median (IQR) was calculated on 17 individuals (6 immunocompetent pts and 11 immunocompromised pts) with more than one HAdV positive sample from stools. *** Percentage was calculated on 58 individuals, 45 immunocompetent pts and 13 immunocompromised pts, with available data. $ Two-sided *p*-values were calculated by Mann–Whitney test, or Chi-square test for trend, as appropriate.

**Table 2:** Univariate and Multivariable Logistic Regression Analysis of Risk Factors Associated with Infection by Adenovirus Species A, B, and C.

	Univariate Analysis		Multivariable Analysis	
Variable	OR (IC 95%)	*p*-Value	OR (IC 95%)	*p*-Value
Age	1.142 [1.024–1.274]	0.017	1.080 [0.871–1.338]	0.483
Male	0.926 [0.323–2.655]	0.926		
Days of hospitalization	1.016 [1.002–1.031]	0.036	0.988 [0.968–1.010]	0.281
Time of positivity for HAdV	1.033 [0.99–1.068]	0.057		
Time of positivity for gastrointestinal HAdV	1.048 [0.995–1.103]	0.074		
Viral load (Ct)	1.467 [1.189–1.811]	<0.001	1.508 [1.161–1.958]	0.002
Immunocompromised status	8.500 [2.246–32.174]	0.002	10.201 [0.307–339.3]	0.129
Gastrointestinal symptoms	0.130 [0.023–0.720]	0.020		
Gastrointestinal viral co-infections	4.00 [0.927–17.522]	0.063		
Fever	6.500 [1.916–22.052]	0.003	12.99 [1.134–148.997]	0.039

OR: Odds Ratio; IC: Confidence Interval; Ct: Cycle threshold.

**Table 3:** Comparison of viral load (VL) according to species and immunological status.

	Overall	Immunocompetent	Immunocompromised	*p*-Value ^$^
Viral Load, Median [IQR] (Ct Values)				
A (N = 4)	16.6 [15.0–17.8]	(N = 1) 16.1 [16.1–16.1]	(N = 3) 17.0 [13.8–18.6]	1.000
B (N = 2)	19.0 [16.0–21.9]	(N = 1) 16.0 [16.0–16.0]	(N = 1) 17.3 [17.3–17.3]	1.000
C (N = 9)	21.0 [20.3–24.3]	(N = 6) 20.6 [19.2–21.1]	(N = 3) 24.3 [24.2–27.4]	**0.024**
F (N = 37)	10.9 [9.2–15.0]	(N = 33) 10.6 [9.1–15.1]	(N = 4) 13.0 [11.1–14.1]	0.620
Total (N = 52)	14.3 [9.7–17.5]	(N = 41) 12.5 [9.3–16.1]	(N = 11) 17.0 [13.1–24.2]	**0.040**

^$^ Two-sided *p*-values were calculated by Mann–Whitney test.

## Data Availability

The 60 HAdV raw sequences obtained in this study are openly available on the European Nucleotide Archive (ENA) under accession n. PRJEB89261. The list of samples and relative accession numbers is available in Table S1.

## Supplementary Materials

The following supporting information can be downloaded at: https://www.mdpi.com/article/10.3390/v17111480/s1, Figure S1: Estimated Maximum Likelihood phylogenetic tree based on the whole-genome sequences of 38 F41 HAdV isolates. Table S1: List of samples and relative ENA accession numbers.

## Abbreviations

The following abbreviations are used in this manuscript:

Ct	Cycle threshold
HAdV	Human Adenovirus
HSCT	Hematopoietic Stem Cell Transplantation
ICTV	International Committee on Taxonomy of Viruses
ML	Maximum Likelihood
SNP	Single Nucleotide Polymorphysm
WGS	Whole Genome Sequencing
