# Supplementary figures and images for: Whole-Genome Sequencing of Adenovirus Genotypes and Clinical Implications in Pediatric Patients
Source: Viruses. 2025 Nov 6;17(11):1480. doi: 10.3390/v17111480 (PMC12656921; doi:10.3390/v17111480)

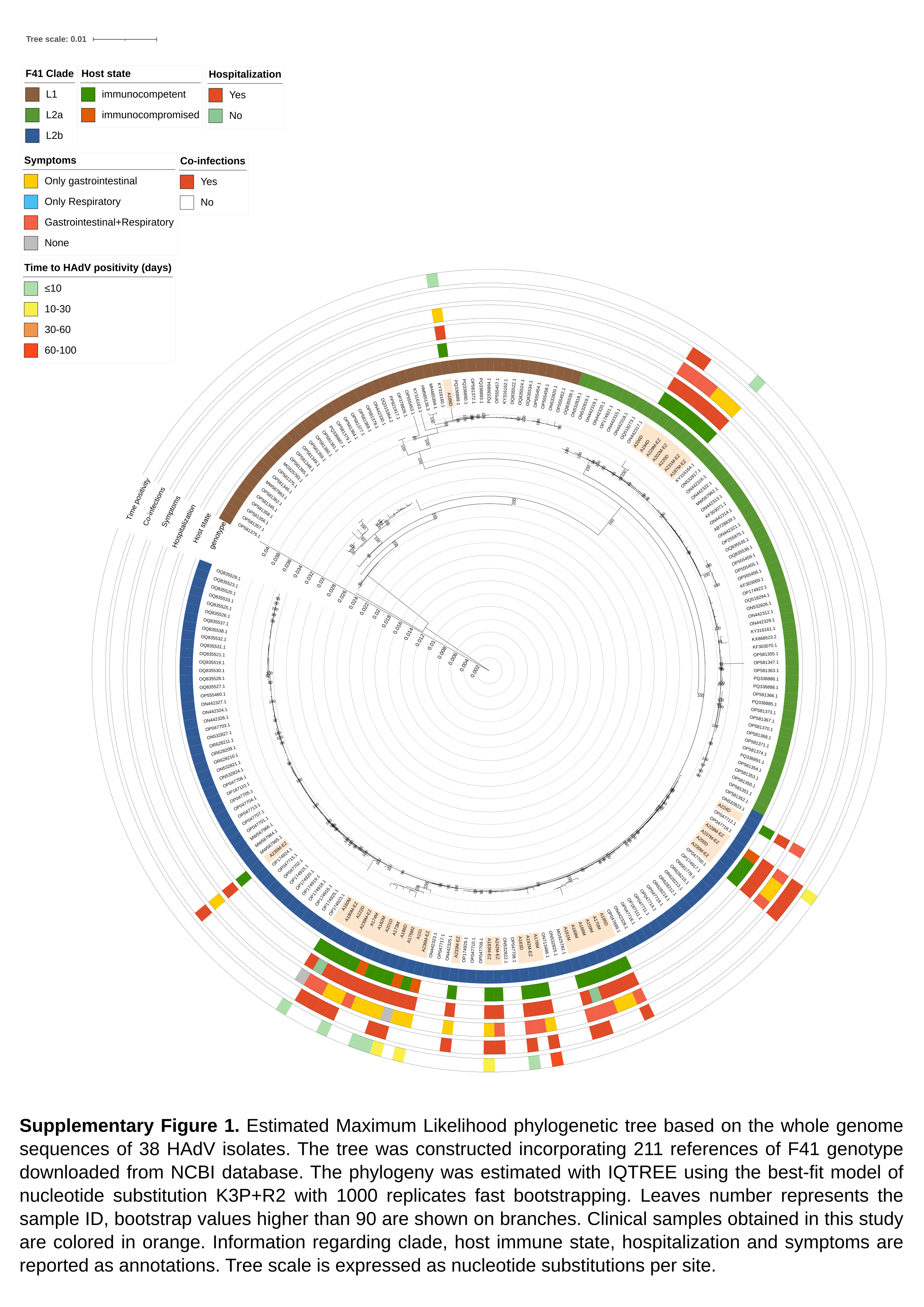

Supplement: Supplementary file 1 [file viruses-17-01480-s001.zip › S1_legend_new.png]
